# Modification of Poly(vinyl chloride) with Bio-Based Cassia Oil to Improve Thermo-Mechanical and Antimicrobial Properties

**DOI:** 10.3390/ma16072698

**Published:** 2023-03-28

**Authors:** Katarzyna Skórczewska, Joanna Szulc, Krzysztof Lewandowski, Anna Ligocka, Sławomir Wilczewski

**Affiliations:** 1 Faculty of Chemical Technology and Engineering, Bydgoszcz University of Science and Technology, Seminaryjna 3, 85-326 Bydgoszcz, Poland; 2 Faculty of Agriculture and Biotechnology, Bydgoszcz University of Science and Technology, Bernardynska 6, 85-029 Bydgoszcz, Poland

**Keywords:** poly(vinyl chloride) PVC, cassia oil, modification

## Abstract

The purpose of this study was to modify plasticised PVC to obtain a material with antimicrobial properties and selected mechanical properties. Natural cassia oil (CO) was used to modify plasticised PVC materials. The modified material was produced by extrusion. The introduced modifier had a maximum concentration of 20 phr. Rheological and mechanical properties were evaluated, and the glass transition temperature was determined. The antioxidant and antimicrobial activity of the agar diffusion method was investigated by analysing the growth inhibition zones against *Enterococcus faecalis* and *Listeria monocytogenes*. A favourable effect of the cassia oil content on the increase in antioxidant activity of the developed polymeric materials was found with an increase in the modifier content and the duration of action (30 days). The largest growth restriction zones were observed for *L. monocytogenes*, i.e., they showed the highest sensitivity to the modified material. The simultaneous decrease in modulus of elasticity, increase in elongation at break, and decrease in *T_g_* indicate that the modifier has a plasticising effect on PVC. The developed material may find application as an active and/or functional material, especially as an emitter of antimicrobial agents, in the packaging used to store minimally processed food.

## 1. Introduction

Currently, materials, especially polymeric materials, with antimicrobial properties are of great scientific and application interest. Extensive research has been conducted on antimicrobial additives of various types, including nanoparticles, as well as on many substances of natural origin, which are characterised by their biodegradability and non-toxicity to humans and the environment, in addition to their high effectiveness in eliminating and/or reducing the growth of pathogenic microorganisms.

Antimicrobial polymeric materials are used wherever it is important to prevent or reduce the growth of unwanted microorganisms, especially in food production, packaging, medical applications, and many other areas, such as construction, public transport, or animal husbandry. In the last few years, these materials have been increasingly researched and sought after [1]. Antimicrobial packaging, an active packaging concept, can be considered an extremely challenging technology that could have a significant impact on shelf-life extension and food safety [2].

Polyvinyl chloride (PVC) is an important thermoplastic with many advantages. Due to its useful properties, its ability to be modified in a wide range of ways, and the fact that its properties remain unchanged over long periods of time, PVC plays an essential role in modern applications, such as building construction, medical applications, and the packaging industry. Due to the high biocompatibility and hemocompatibility of PVC [3], this polymer is the most widely used plastic in healthcare, accounting for approximately 25% of all medical-plastic compounds. A common way of modifying PVC is through the use of plasticisers [4,5,6], resulting in materials with a broad range of flexibility and transparency and a reduced processing temperature. Plasticised PVC grades are used for the production of hoses and cable insulation, liners, and all kinds of films, e.g., for packaging and blood bags.

The imparting of antibacterial properties to PVC can be carried out by surface application of biocides or by surface release. Antibiotics, such as trixolate, have been used for this purpose [7], as have nanoparticles, e.g., silver with quercetin [8], surface covalently bonded guanidine-based antimicrobial polymers [9], or aluminium alkoxides [10]. These solutions, however, did not ensure a reduction in the accumulation of antimicrobial agents in the environment. Increasingly, the search for new solutions is based on compounds of natural origin that are capable of biodegradation.

A number of natural substances with antimicrobial activity that can potentially be used in polymer modification are known. One of these is essential oil. Essential oils are complex mixtures of volatile active compounds and aromatics, and their microbiological activity depends primarily on their content of terpenes or monoterpenes and phenolic or aromatic compounds [11]. There are many edible and medicinal plants with high antimicrobial effects, such as thyme (*Thymus vulgaris* L.), tea (*Camellia sinensis* L.), garlic (*Allium sativum* L.), turmeric (*Curcuma longa* L.), berries belonging to the Rosaceae family, and cinnamon (species belonging to the Cinnamomum genus) [12]. 

Currently, scientists are interested in the use of different types of essential oils and substances extracted from them to modify polymers that can be used in food packaging [13,14,15]. Natural antimicrobial agents include extracts from spices such as cinnamon, allspice, clove, thyme, rosemary, and oregano, and other plant extracts such as onion, garlic, radish mustard, and horseradish [2] A number of methods of introducing essential oils into polymeric materials are being investigated. The essential oil can be incorporated into a polymer matrix or applied to a polymer film surface [16]. The following methods are known in the literature for obtaining modified material: the cast solution method [17], the encapsulation method from a polymer solution [18,19], or film preparation by electrospinning [20]. Multilayer films are also produced using the solution casting method [21]. A limitation in the use of essential oils to modify polymers in the molten state may be the insufficient thermal stability of these active substances during processing. Therefore, polymers with reduced processing temperatures, e.g., a mixture of PLA and PBAT with cinnamon oil, which was processed at 160 °C, were used in the extrusion modification of polymers [22].

To date, there is little information in the literature regarding the modification of PVC with essential oils. Some works consider essential oils to react chemically as plasticisers [23,24] or PVC stabilisers [25]. To modify PVC with essential oils, clove oil [26,27], orange essential oil [28], zinc nanoparticles with pistachio green hull essential oil [29], and *Moringa oleifera* oil with Ag nanoparticles [30] were used. 

More attention is also being given to essential oils obtained from plants of the genus Cinnamomum. One of these is cassia oil, which is commonly used as a spice, in aromatherapy, or in medicine. It has shown wide varieties of biological functions, such as antioxidant, anti-inflammation, anti-tumor activity, antimicrobial, and anti-diabetic effects. Cassia oil as a secondary metabolite is due to its excellent antibacterial effects against both Gram-positive and Gram-negative bacteria [31]. Various types of essential oils derived from Cinnamomum oil are used for the preparation of active packaging [32,33,34,35,36].

Cassia oil, unlike cinnamon oil, is extracted from the fragrant cinnamon tree (*Cinnamomum cassia*) and has a higher content of cinnamaldehyde in comparison. The content and proportion of constituents varies depending on the method of extraction, as well as the part of the plant and the location of cultivation [37,38,39]. The major components identified in cassia oil were (E)-cinnamaldehyde (92.0–98.0%), (Z)-cinnamaldehyde (0.8–2.7%), β-caryophyllene (0.4–3.6%), coumarin (0.1–1.6%), and α-ylangene (0.1–2.7%) [40]. 

The use of cassia oil to modify PVC and obtain a material with antimicrobial properties has not yet been reported in the literature. In our study, plasticised PVC materials with cassia oil with favourable antimicrobial properties and suitable mechanic and thermal characteristics were developed, which could be used as packaging materials in the food industry to extend product shelf life.

## 2. Materials and Methods

### 2.1. Materials

Polyvinyl chloride S-61 (PVC) was purchased from Anwil S.A. (Poland). A thermal stabiliser, Reagens CLX/743, was purchased from Reagens S.P.A. (Italy). A plasticizer (ES), an epoxidized soybean oil, was purchased from Boryszew—Erg S.A. (Poland). Cassia oil (CO) and 2,2-Di(4-tert-octylphenyl)-1-picrylhydrazyl (DPPH*) were purchased from Sigma-Aldrich (Germany/USA). Ethanol (96%) and methanol were purchased from Avantor S.A. (Poland). All the chemicals were of an analytical grade. Demineralized water was used for all these experiments. 

The microorganisms used in the research came from the collection at the Universität Hohenheim Institut für Umwelt- und Tierhygiene sowie Tiermedizin mit Tierklinik and the collection of pure cultures from the Department of Microbiology and Food Technology at the Bydgoszcz University of Science and Technology. In studies on the impact of the bacteriostatic properties of cassia oil, the following bacterial species were tested: *Listeria monocytogenes* and *Enterococcus faecalis*. The media used in the microbiological tests were enriched broth (Merck 07882) and nutrient agar (Merck 107881) purchased from Merck (Germany).

### 2.2. Sample Preparation

The PVC mixtures were prepared in a Z-shape blade mixer (FDO 234H, Brabender GmbH & Co. KG, Duisburg, Germany). The temperature of the bowl was 85 °C, and the speed of the mixer was 56 min^−1^. PVC (100 phr) and thermal stabiliser (4 phr) were introduced into the kneading trough and mixed for 3 min. A mixture of plasticiser (50 phr) and cassia oil (from 0 phr to 20 phr) was then added in a small stream and stirred for a further 10 min. Finally, dry, free-flowing, non-agglomerated PVC dry blends were obtained.

The dry blends obtained were extruded using a laboratory single-screw extruder (Brabender GmbH & Co. KG, Duisburg, Germany) (screw diameter = 15 mm, L/D = 13), through a cylindrical nozzle with a diameter of 3 mm and a length of 40 mm. The processing temperatures were: hopper—18 °C; 1st zone—130 °C; 2nd zone—160 °C; head—165 °C. The screw speed was 40 min^−1^. The extrudate obtained was cooled in air and granulated. 

The materials obtained were used to produce 2 mm × 200 mm × 200 mm and 1 mm × 200 mm × 200 mm plates by pressing. A frame and plates of polished stainless steel were used as the pressing mould. The processing parameters were: temperature 160 °C, maximum pressing pressure 5 MPa, and total processing time 4 min. 

The PVC materials produced were coded as follows: for example, a sample labelled PVC 50ES 20CO denotes PVC matrix material containing 50 phr plasticiser and 20 phr cassia oil.

### 2.3. Material Characterizations

#### 2.3.1. Determination of Processability 

Determination of processability was carried out during the processing of blends in a Brabender plastographometer chamber (Brabender GmbH & Co. KG, Duisburg, Germany) while recording changes in the torque and temperature of the kneaded material over time. The processing conditions were: chamber temperature 165 °C, main rotor speed 30 min^−1^, chamber free volume 50 cm^3^, processing time 20 min, and a constant charge mass of 55 g. The processed material temperature (T) and torque (M) values were determined after 2 min, 10 min, and 20 min of kneading.

#### 2.3.2. Determination of Rheological Properties

The rheological properties of the materials obtained were determined using a Dynisco LCR 7001 capillary rheometer (Franklin, MA, USA). The measurement temperature was 165 °C. The material was melted in the cylinder of the rheometer for 5 min before measurement. A cylindrical nozzle with a diameter of 2 mm and a length of 40 mm was used. The pressure of the molten material before entering the measuring nozzle was determined at a specific volumetric flow rate corresponding to an apparent shear rate in the range from 15 s^−1^ to 912 s^−1^. The Rabinovitch-corrected viscosity of the molten polymer under measurement conditions was determined according to the methodology described in [41,42,43].

#### 2.3.3. Determination of Mechanical Properties

The mechanical properties of static tension were determined in accordance with EN ISO 527 by using the Zwick Roell Z010 universal testing machine (Zwick GmbH & Co. KG, Ulm, Germany). Standardised test specimens (type 1 BA) were cut from 2 mm-thick tile plates with a blanking tool. The test speed was 1 mm/min for the determination of the modulus of elasticity and 50 mm/min for other parameters. The modulus of elasticity E_t_, tensile strength σ_M,_ and the elongation at break ε_B_ were determined.

#### 2.3.4. Determination of the Glass Transition Temperature

The tests were carried out using a DMA Artemis device (Netzsch GmbH & Co. Holding KG, Selb, Germany). The determination was made in a three-point bending system with a strain of 10 μm and a frequency of 1 Hz. The temperature range was −100 °C–100 °C with a 2 °C/min increase rate. The relatively small amplitude and low strain frequency allow for a measurement in a linear viscoelastic range with a low value of the loss modulus [44]. The glass transition temperature (*T_g_*) was determined based on changes in the value of the storage modulus (E′) as a function of temperature at the beginning of a rapid decrease in value E′ (*T_g_* onset) [45]. From the DMA thermograms obtained, the maximum values of the loss modulus (E″) and tan δ were established. The stiffness of material based on E′ was also determined at −25 °C, 0 °C, and 25 °C. 

#### 2.3.5. Fourier-Transform Infrared Spectroscopy (FTIR) Analysis

Material samples were analysed by Fourier-transform infrared spectroscopy (FTIR). The study was carried out using an Alpha instrument (Bruker Optics GmbH & Co. KG, Ettlingen, Germany), using the ATR (reflectance) technique, in the range 4000–500 cm^−1^, with a resolution of 4 cm^−1^. The cassia oil content of the polymeric materials was assessed.

#### 2.3.6. Determination of Thermal Properties by Thermogravimetric Analysis

Thermogravimetric measurements were made with a TG 209 F3 (Netzsch GmbH & Co. Holding KG, Selb, Germany) device in nitrogen. The measurement was carried out in the temperature range of 30–900 °C with a temperature change rate of 10 °C/min. The investigated sample mass ranged from 15–20 mg, with a gas flow rate of 30 mL/min. The losses of 1% (T_1_), 5% (T_5_), and 50% (T_50_) of the sample mass, the temperature of the maximum decomposition rate (T_DTG_), and the residue after decomposition at 900 °C (RM) were determined. 

#### 2.3.7. Determination of Antioxidant Activity 

DPPH* radical scavenging activity was determined using the method of Brand-Williams with a slight modification [46]. Polymer samples of 10 mm × 10 mm × 2 mm were cut out of the materials. Each sample was placed in a 50 cm^3^ conical tube with a cap and mixed with 9 cm^3^ of distilled water. Samples were stored for 30 days at room temperature without access to light. The obtained supernatant was analysed for DPPH* radical scavenging activity after storage for 1 h, 10 days, and 30 days.

The supernatant (1.0 cm^3^) was mixed with 3.0 cm^3^ of 0.5 mM 2,2-diphenyl-1-picryl hydrazyl (DPPH*) in methanol. The mixture was mixed vigorously with the vertex mixture and allowed to stand at room temperature in the dark for 15 min. The absorbance of the resulting solution was measured at 517 nm using a spectrophotometer (Agilent 8453 UV-visible Spectroscopy System, USA). The sample blank was prepared in the same manner, except methanol was used instead of DPPH* solution. 

#### 2.3.8. Determination of the Antimicrobial Activity

The agar diffusion method was used to determine the antibacterial activity of PVC materials. Polymer discs (10 mm diameter, 2 mm thick) were placed on the surface of the nutrient agar dishes with microorganisms. The experiment used 24-h cultures of *L. monocytogenes* and *E. faecalis* on liquid media (enriched broth, Merck 07882). 0.1 cm^3^ of each suspension was taken and spread on the surface of a standard I nutrient agar (Merck, 107881). The agar plates with discs were incubated at 37 °C for 24 h. The tests were carried out in triplicate. The inhibition zones were observed.

PVC materials were also used as emitters. PVC-cassia oil samples and spinach leaves contaminated with a suspension of *L. monocytogenes* and *E. faecalis* of known cell densities were placed in food-contact plastic containers. The container with one 20 mm × 20 mm × 2 mm sample cut from the material containing 0 phr, 10 phr, and 20 phr of CO and one contaminated spinach leaf was covered with plastic foil and stored at 4 °C for 14 days. The level of spinach leaf bacterial contamination was tested on a selective medium after 24 h, 48 h, 72 h, 7 days, and 14 days of storage. The spinach leaf was immersed in 9 mL of a 0.85% NaCl solution and mixed well for 5 min. Next, 1 mL of bacterial suspension was diluted in series 10^−1^–10^−6^. Tenfold serial dilutions were incubated at a temperature of 37 °C for 24–48 h. After incubation, the colonies were counted.

## 3. Results

### 3.1. Determination of Processability and Rheological Characteristics

**Figure 1:**
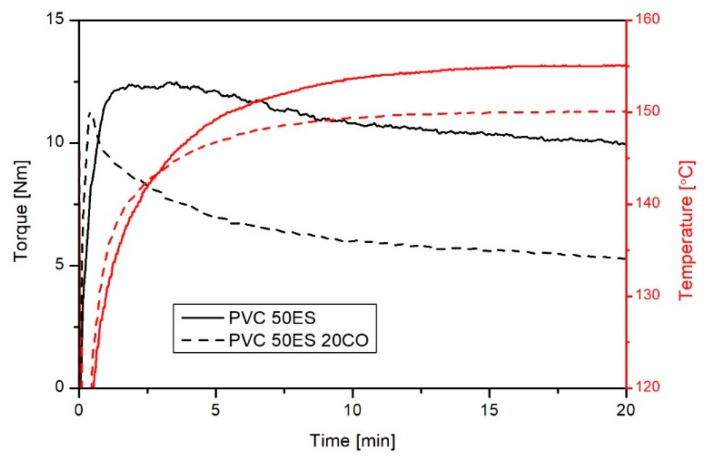
An exemplary plastogram of PVC 50ES and PVC 50ES 20CO.

Based on plastographometric analyses, the effect of cassia oil on the processing properties of plasticized PVC was assessed. Examples of PVC 50ES and PVC ES 20CO processing plastograms are shown in Figure 1, while selected values of torque and temperature of the material for different kneading times are presented in Table 1. 

Plastograms are characteristic of PVC with a large share of plasticizer. Fusion takes place immediately after loading the plastographometer chamber [6,47,48]. The addition of CO significantly changes the processing properties of PVC blends. In the first few minutes of kneading the modified mixtures, a faster increase in torque is observed, which proves faster fusion and homogenization of the processed material. The determined value of the torque decreases after 2 min with the increase in the CO content, while the temperature of the mixture is higher than in the absence of the active agent. The increase in temperature at this stage of processing is the result of increased heat exchange and better homogenization of the processed material due to its lower viscosity. As the kneading time increases, the torque and temperature of the material stabilise. At the end of kneading, a clear effect of CO on the reduction in PVC viscosity was found, which is evidenced by a large reduction in torque. Along with the change and thinning of the viscosity, the heating effect of the material decreases as a result of reduced energy dissipation from the internal friction of the material [49,50]. Such results are characteristic of PVC plasticizing additives [6].

The course of kneading does not indicate the volatilization of cassia oil or the degradation of the processed materials during processing. It should be accompanied by an increase in viscosity and an increase in torque at the end of processing. Based on plastographometric tests, it was found that plasticised PVC modified with cassia oil can be processed by conventional processing methods.

**Figure 2:**
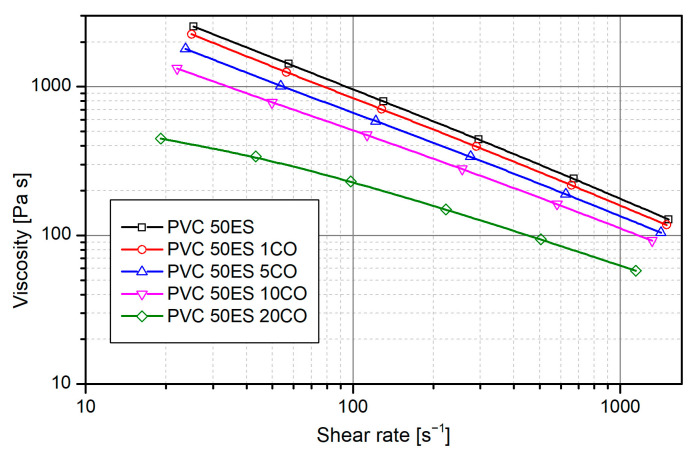
Viscosity curves for materials with different cassia oil contents.

Processing properties of the produced materials were also determined based on rheological tests. Materials produced in the extrusion stage were tested. Viscosity curves determined for the obtained materials are shown in Figure 2. 

The viscosity curves are characteristic of polymers in a molten state. The melted polymer material belongs to non-Newtonian shear-thinning fluids. As the shear rate increases, the viscosity decreases significantly. As in the case of plastographometric tests, a significant decrease in viscosity was found with an increase in the share of cassia oil in the materials. This is the effect of reduced interactions between polymer macromolecules, which is characteristic of plasticizing modifiers. Reducing the viscosity of the polymer in terms of technology brings many benefits. First, reduced flow resistance makes the processing process easier and reduces the risk of material self-heating at high shear rates. It is extremely important when processing polymers sensitive to high temperatures, such as PVC. The pronounced plasticizing effect of cassia oil also allows the processing to be carried out at lower temperatures, which in turn will limit the degradation not only of the polymer matrix but also of the active agents contained in CO.

### 3.2. Mechanical Analysis

**Figure 3:**
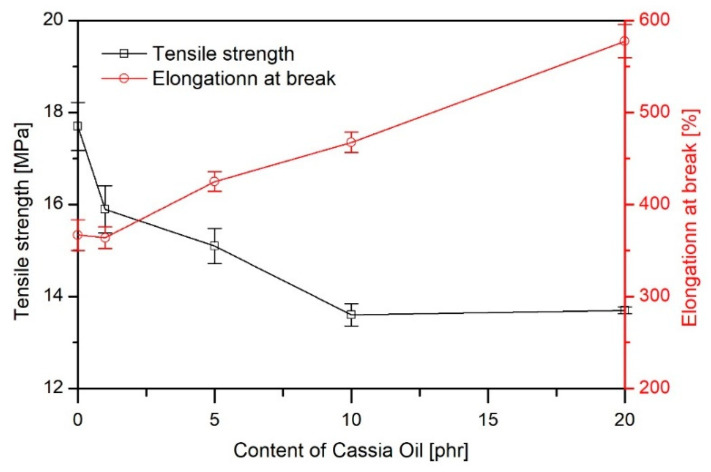
Tensile strength and elongation at break as a function of CO in the PVC matrix.

**Figure 4:**
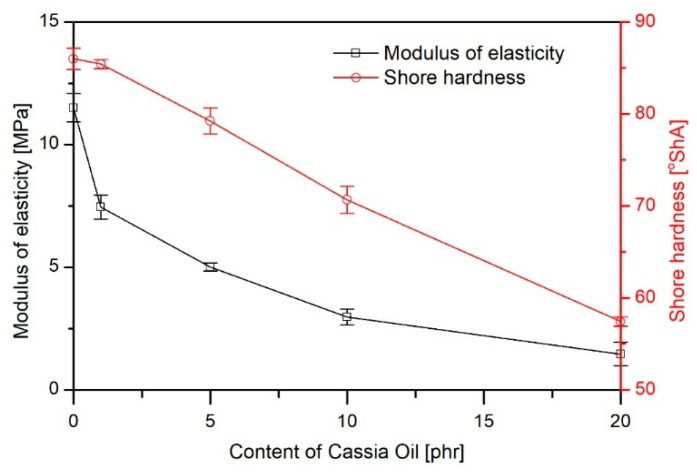
Modulus of elasticity and Shore hardness as a function of CO in the PVC matrix.

Figure 3 shows the dependence of tensile strength and elongation at break, while Figure 4 presents the modulus of elasticity and hardness in relation to the content of CO in the polymer material. 

As the proportion of CO in the material increased, a decrease in tensile strength was observed (from 17.7 MPa for PVC 50ES to 13.7 MPa for PVC 50ES 20 CO) with an increase in *ε_B_*. At the same time, a clear increase in elasticity was found. This is evidenced by a decrease in both *E_t_* and hardness values. This is characteristic of materials modified with plasticising additives [6,28].

### 3.3. Thermomechanical Analysis DMA and T_g_ Determination

**Figure 5:**
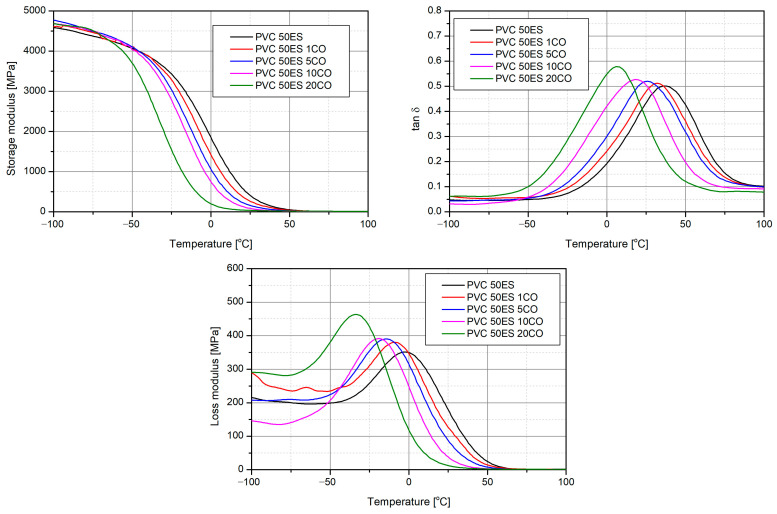
Thermograms of the DMA analysis of investigated materials.

One way to assess the plasticising effect of a substance on a polymer matrix is by observing the reduction in the glass transition temperature. Based on the DMA analysis, the glass transition temperature values of the tested materials were determined. The thermograms of DMA are presented in Figure 5, and the characteristic values are summarised in Table 2. 

As the content of this additive increased, the value of the glass transition temperature decreased. The CO-free material was characterised by a *T_g_* value of −25.7 °C, while the introduction of 20 phr CO reduced the *T_g_* to −51.9 °C. The values of E′, E″, and tan δ at a given temperature also decrease with an increase in CO content. These observations once again point to the plasticising effect of CO. Substances with a plasticising effect reduce the glass transition temperature and modulus of elasticity [4,6].

### 3.4. Thermogravimetric Analysis by TGA Method

**Figure 6:**
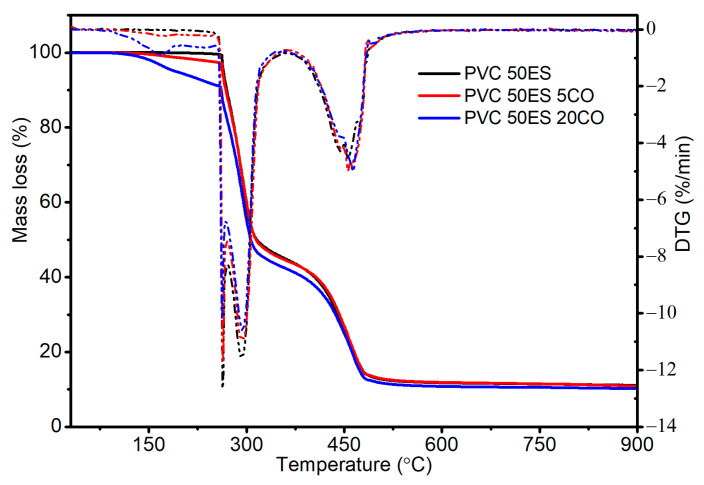
TGA (solid curve) and DTG (dotted line) thermograms of selected PVC materials.

The decomposition of PVC 50ES occurs in two stages, while that of PVC 50ES with CO occurs in three stages (Figure 6). In the first stage, in the temperature range of 30–180 °C with a maximum T_DTG1_ of approx. 169 °C, the weight loss correlates with the proportion of CO, as confirmed in the literature [18,19,51]. 

The weight loss values for the individual materials are 0.15–1.5% lower than would be indicated by the original amount of CO in the prepared PVC dry blends (Table 3). This indicates that the processing conditions allowed CO to be retained in the polymer matrix without significantly decreasing its concentration in the obtained polymeric materials. The small differences in the determined values are mainly due to the overlapping of subsequent stages of mass loss associated with the degradation of the remaining components of the material or may be due to the loss of the active agent during the processing steps.

In the second stage of decomposition, in the temperature range of 200–300 °C, a very intensive initiation of the destruction of the plasticised PVC matrix occurs. The observed T_DTG2_ maximum of approx. 261 °C is associated with an intensive decomposition of plasticiser, while the T_DTG3_ maximum of approx. 293 °C is associated with the dechlorination of PVC macromolecules [45,52]. The next stage above 400 °C is attributed to the crosslinking of chains containing C=C bonds, as the process of thermal degradation of polyenes involves cyclization and the splitting of chains [53]. 

The values at which 1%, 5%, and 50% sample weight loss occurs were determined. The decrease in T_1_ values with increasing CO content in the matrix is due to the degradation or evaporation of the antimicrobial agent. The T5 value is comparable when the concentration of CO is up to 10 phr and is related to the initiation of PVC degradation. In the case of PVC 50ES 20CO, the lower T_5_ value is due to the loss of CO. The T_50_ of the materials tested, regardless of CO content, is comparable to that obtained for the unmodified matrix. The release of the active substance at a temperature as low as 30 °C suggests the possible use of the developed modified materials as emitters.

### 3.5. FTIR Analysis

**Figure 7:**
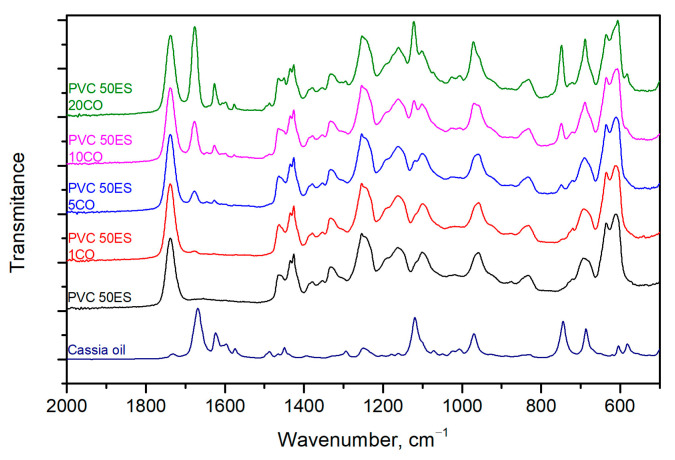
FTIR spectra of obtained materials in the wavenumber range 2000–500 cm^−1^.

Cassia oil, similar to other essential oils, is a multicomponent mixture, which makes it very difficult to identify the individual components [54]. According to the literature, there may be such compounds as cinnamaldehyde, β-caryophyllene, coumarin, and α-ylangene [40]. Therefore, the analysis of CO by FTIR was based on the identification of functional groups through the location of the different bands on the spectrum. FTIR spectra were taken in the range of 4000–500 cm^−1^, but detailed analysis was performed on the fingerprint region in the range of 2000–500 cm^−1^ (Figure 7). In the FTIR spectra of CO, strong peaks appear at about 1670 cm^−1^ (C=O and C=C stretching) and 1120 cm^−1^ (C-O stretching). The presence of the phenyl group is confirmed by the peaks at 1600 cm^−1^, 1580 cm^−1^, and 1450 cm^−1^, which correspond to frame vibrations of the benzene rings, and the strong peaks at 740 cm^−1^ and 690 cm^−1^, which correspond to bending vibrations of =CH on the aromatic rings [55]. 

In the wavelength range analysed, a peak at around 1430 cm^−1^ (C-H bonds in the PVC chain), a peak at 1252 cm^−1^ (bending vibrations of the C–H originating from CHCl), and the bands at 1099 cm^−1^ and around 680 cm^−1^ (as a result of the presence of C–C and C–Cl groups) were observed [56].

The presence of an ES plasticiser is indicated by a peak at 1746 cm^−1^, which corresponds to the aliphatic -C=O stretching of esters, and a peak around 820 cm^−1^ that corresponds to an epoxy group [56,57]. The other peaks described in the literature [58] that are characteristic of ES overlap with the bands observed for the polymer material components.

**Figure 8:**
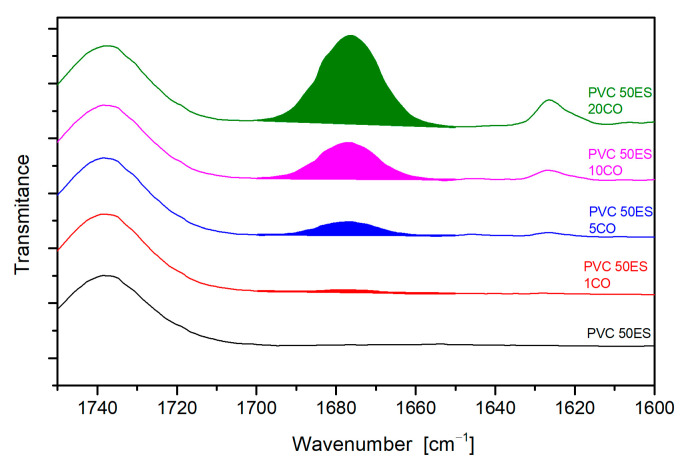
Comparison of the proportions of the active ingredient in materials based on band area analysis at wavenumbers from 1700 cm^−1^ to 1650 cm^−1^.

In addition, band characteristics for the PVC matrix also partly overlap with bands indicating the presence of CO in the matrix. However, the FTIR spectra of modified materials show separated bands, which can be associated with the presence of CO in the matrix, i.e., 1670 cm^−1^, 1620 cm^−1^, and 740 cm^−1^. In order to verify the presence of CO in the matrix, FTIR spectra were compared, and the area of the 1670 cm^−1^ band was determined as an indicator of changes in the amount of CO in the PVC matrix, as shown in Figure 8. As can be observed, with an increase in the proportion of CO in the matrix, the area under the studied peak increases.

**Figure 9:**
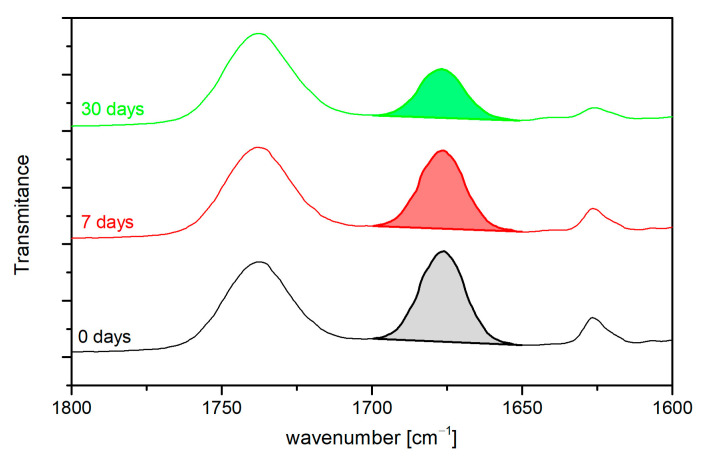
Analysis of peak area changes at 1700 cm^−1^–1650 cm^−1^ of the PVC 50ES 20CO sample as a function of migration time (0, 7, and 30 days).

The determined correlation between the peak area and changes in CO concentration in the material was used to assess the possibility of using the developed polymer material as an emitter. Samples in the form of a disc were kept in an air-conditioned room at 25 °C, and FTIR measurements were carried out after 7 days and 30 days. From the analysis of the spectra, the time-dependent change in CO concentration was determined, as exemplified in Figure 9.

The CO content decreases with time, as indicated by a decrease in the area under the analysed peak. On this basis, the migration of CO per g of polymeric sample into the air was determined as a function of the material used and the test time (Table 4). The migration values obtained increase with the concentration of CO in the material and with increased migration time. CO migration is caused by weaker interactions with PVC macromolecules than common plasticisers, which show a higher affinity for PVC macromolecules due to strong dipole-dipole interactions. In the case of the material under development, this is an advantage due to its constant ability to release the active agent based on CO migration to the surface.

Furthermore, this process depends on many parameters, such as the initial concentration of the active substance, temperature, time, and the type of medium into which it migrates. In the work [46], PVC materials modified with orange essential oil were evaluated for migration into different media: 10% ethanol, olive oil, and water (162 h). Migration was observed in all media, with the strongest migration to olive oil due to the affinity and solubility between them.

The results obtained confirm its ability to release the natural active ingredient over the tested time period (30 days) and thus the likely prolonged microbiological action of the developed material. This confirms the assumed ability of the material to not only have a microbiological contact effect but also to modify the atmosphere of, for example, packaged food, i.e., act as an emitter. 

These results made it possible to design an experiment to evaluate the effect of the emitter on microbiological quality, expressed as the effectiveness of inhibition of microbial growth occurring on minimally processed foods.

### 3.6. Antioxidant Activity

Testing the antioxidant properties of the material is important if it is assumed that the described PVC with the addition of cassia oil will be dedicated to the food industry. Oxidation processes are undesirable in food processing, especially during the storage of food products. The occurrence of oxidation reactions in food, especially with lipids and proteins, is related to unfavourable changes in the sensory attributes of the product (e.g., rancidity and changes in colour and texture) and is responsible for quality decay and economic losses [59]. In addition to food changes, lipid oxidation generates harmful compounds that have been linked to several human diet-related diseases, including atherosclerosis, cancer, inflammation, and ageing processes, among others [60].

These reactions support the introduction of substances with antioxidant properties into the food itself and/or packaging in order to limit or inhibit the harmful transformations of food ingredients. During storage, the tested material released compounds that have antioxidant properties and stabilise the free radical DPPH* (Table 5). Antioxidant activity was observed for the control PVC material. This is related to the presence of the plasticiser and stabiliser, which also manifest slight antioxidant effects due to their structure. The higher concentration of CO shows higher antioxidant activity. This dependence is also shown in FTIR analysis (Figure 9) and material mass change during storage (Table 4). 

Material with 20 phr of incorporated cassia oil after 30 days of storage in water is characterised by its higher capacity to scavenge the free radical DPPH* (almost 75%). Szulc et al. [16] demonstrated similar behaviour of PVC material with incorporated thymol, and Braga et al. [8] described the consumption of DPPH* by PVC-based films combined with quercetin and Ag nanoparticles. High antioxidant activity was observed in the PVC-quercetin film after 24 h (60.44%). Quercetin is a strong antioxidant agent, and this may be the reason why PVC films with this compound show a high ability to scavenge the free radical DPPH* in a shorter time. The slow release of active substances with antioxidant properties, which are present in PVC-CO materials, indicates that this material might be dedicated as an active packaging material or as an emitter for food with a long shelf life.

### 3.7. Antimicrobial Activity

The antimicrobial effect of essential oils of Cinnamomum has been described in many papers [61,62,63,64,65].

**Figure 10:**
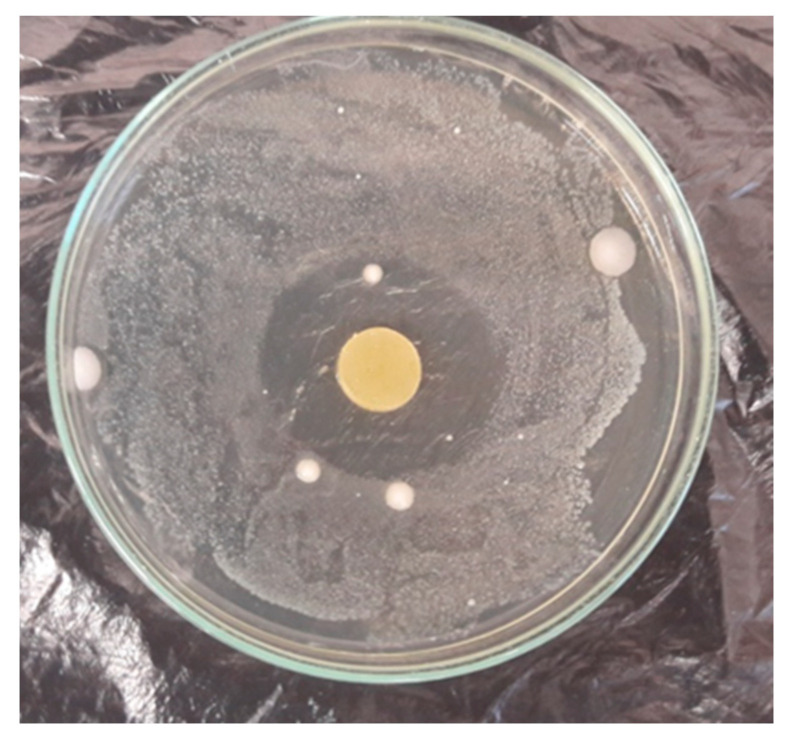
*L. monocytogenes* inhibition zone of PVC 50ES 10CO.

The antimicrobial activity of the material has been confirmed, but there is still little information about the effect of CO when it is introduced into the polymer matrix. As can be seen in Table 6, the antibacterial activity of the material increased with the increase in CO concentration in the polymer, but only in the range of 5–20 phr. The highest concentration (20 phr) did not cause the expected increase in the zone of inhibition of bacteria. In this case, the inhibition zone was slightly smaller than the material with 10 phr of CO. *Listeria monocytogenes* (Figure 10) turned out to be the most sensitive to PVC-CO material. The antimicrobial activity of the material with starch and cinnamon oil was demonstrated by Souza et al. [66]. The zones of inhibition they obtained showed that the material with cinnamon bark oil is effective in limiting the growth of pathogenic microorganisms.

**Figure 11:**
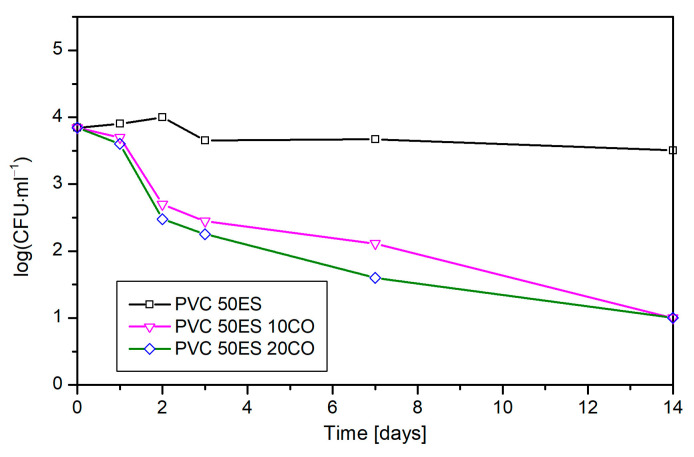
Influence of cassia oil concentration in PVC material on the growth of *L. monocytogenes* present on a spinach leaf.

Considering the antimicrobial properties of cassia oil [67,68], as well as its effect on pathogens in contact, proven by the agar diffusion test, it can be expected that the introduction of antimicrobial substances, such as essential oils, into the matrix of the packaging or into the appropriate emitters may significantly affect the presence of pathogenic microflora. This property is important for the food industry, especially for packed food, and introducing this solution to the industry can increase food safety. In addition, the conducted migration tests (Table 4) to determine the weight loss of PVC-CO material during storage confirm the validity of the assumption that the described material can be used as an emitter of substances with antimicrobial properties. This has also been proven in the minimally processed food experiment. The experiment results indicate that PVC-CO materials are good emitters of antibacterial agents because they limit the growth of both tested bacteria. In the case of *L. monocytogenes*, the inhibitory effect of CO already began to show on the second day of the experiment, when the bacterial population was reduced by an average of 1.4 logs (Figure 11). During storage, an increase in the inhibition effect was observed. Material with 20 phr of CO is the most effective at inhibiting the growth of bacteria. On day 14 of storage, the presence of the PVC-CO material caused a 2.5 log decrease in the bacterial count compared to the control sample. 

**Figure 12:**
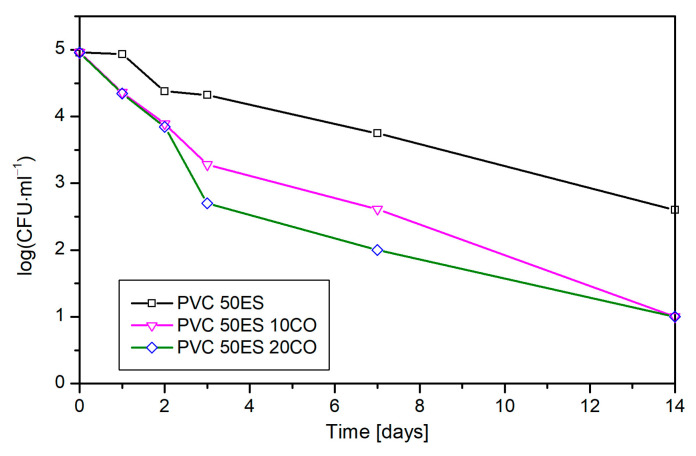
Influence of cassia oil concentration in PVC material on the growth of *E. faecalis* present on a spinach leaf.

The significant reduction in the CFU of streptococci caused by cassia oil was observed on the third day of the experiment and amounted to 1–1.8 log, depending on the CO concentration. Bacterial reduction remained at a similar level during storage. After 14 days, the material with 10 phr and 20 phr of cassia oil caused the same inhibitory effect, reducing *E. faecalis* populations by 1.6 logs (Figure 12).

## 4. Conclusions

Cassia oil has a plasticising effect on PVC, which was found based on the results of mechanical properties, glass transition temperature, assessment of processing, and rheological properties.

The developed procedure for obtaining modified PVC does not result in a significant loss of active agent in the obtained material. 

With the increase in CO concentration in the PVC material, the ability to deactivate free radicals, which are one of the elementary causes of food quality decay, increases.

The investigation of antimicrobial properties showed the developed PVC materials’ effectiveness in limiting the growth of selected pathogenic microorganisms.

Modified PVC materials are characterised by the migration of the active agent into the air, thanks to which they can be used as emitters of active substances in packaging, especially in the food industry.

It was found that the developed PVC materials could be used as emitters, limiting the growth of selected pathogenic and food-spoiling microorganisms on minimally processed foods, which contributes to extending their shelf life.

## Figures and Tables

**Table 1:** Analysis of plastographometric characteristics.

Material	Kneading Time					
	2 min		10 min		20 min	
	M, Nm	T, °C	M, Nm	T, °C	M, Nm	T, °C
PVC 50ES	12.2	137.0	11.0	153.0	10.1	155.0
PVC 50ES 1CO	11.9	142.2	10.3	153.9	9.5	155.0
PVC 50ES 5CO	11.6	142.7	9.2	153.4	8.6	154.1
PVC 50ES 10CO	10.3	142.7	8.3	151.4	7.6	152.1
PVC 50ES 20CO	8.3	140.9	6.2	149.4	5.4	150.1

**Table 2:** Characteristic values of the DMA analysis.

Material	*T_g_*, °C	Max E″, °C	Max tan δ, °C	E′, MPa		
				−25 °C	0 °C	25 °C
PVC 50ES	−25.7	−2.1	36.9	3433	1867	411
PVC 50ES 1CO	−29.1	−8.9	31.8	3288	1431	264
PVC 50ES 5CO	−36.1	−13.7	25.3	3039	1073	160
PVC 50ES 10CO	−44.0	−19.2	17.9	2790	759	86
PVC 50ES 20CO	−51.9	−33.0	6.6	1551	204	37

**Table 3:** The TGA analysis results.

Material	T_1_, °C	T_5_, °C	T_50_, °C	T_DTG1_, °C	Mass Loss 30–180 °C, %	T_DTG2_, °C	T_DTG3_, °C	RM at 900 °C, %
PVC 50ES	262.3	264.4	315.8	-	0.1	263.5	292.7	11.1
PVC 50ES 1CO	260.6	264.5	316.3	-	0.5	262.9	292.6	10.8
PVC 50ES 5CO	177.5	262.1	313.5	167.8	2.8	262.6	292.7	10.8
PVC 50ES 10CO	153.9	260.4	310.6	169.2	4.9	261.9	293.1	10.8
PVC 50ES 20CO	132.5	190.1	303.5	169.9	9.9	261.3	293.0	10.2

**Table 4:** Migration of active substances to the air.

Material	Migration, mg/g	
	7 Days	30 Days
PVC 50ES 1CO	0.17	3.73
PVC 50ES 5CO	4.62	8.33
PVC 50ES 10CO	10.19	17.74
PVC 50ES 20CO	12.23	38.57

**Table 5:** Radical DPPH* scavenging capacity results.

Material	Antioxidant Activity during the Storage, %		
	0 Days	10 Days	30 Days
PVC 50ES	23 ^bB^	28 ^aD^	31 ^aC^
PVC 50ES 1CO	29 ^cB^	41 ^bC^	47 ^aB^
PVC 50ES 5CO	29 ^bB^	42 ^aC^	49 ^aB^
PVC 50ES 10CO	44 ^bA^	50 ^aB^	53 ^aB^
PVC 50ES 20CO	40 ^cA^	59 ^bA^	74 ^aA^

^a–c^—average values in lines denoted with the same letters do not differ statistically significantly at *p* < 0.05. ^A–D^—average values in columns denoted with the same letters do not differ statistically significantly at *p* < 0.05.

**Table 6:** Influence of different CO concentrations in PVC material on the bacterial inhibition zone.

Material	Bacterial Inhibition Zone Width, mm:	
	Enterococcus Faecalis	*Listeria monocytogenes*
PVC 50ES	0	0
PVC 50ES 1CO	0	3.0
PVC 50ES 5CO	1.0	8.0
PVC 50ES 10CO	2.0	11.0
PVC 50ES 20CO	1.0	9.0

## Data Availability

The data supporting this study’s findings are available from the corresponding authors (Katarzyna Skórczewska and Krzysztof Lewandowski) on request.
